# Effect of Encapsulated Ferrous Sulphate Fortified Salt on Hemoglobin Levels in Anemic Rats

**DOI:** 10.3390/foods11121795

**Published:** 2022-06-17

**Authors:** Dasharath B. Shinde, Santosh S. Koratkar, Vinay Rale, Shashikala NM, Neetu Mishra

**Affiliations:** 1 Symbiosis School of Biological Sciences, Symbiosis International Deemed University, Lavale, Pune 412115, Maharashtra, India; dasharath.shinde@ssbs.edu.in (D.B.S.); santosh.koratkar@ssbs.edu.in (S.S.K.); vinayrale@gmail.com (V.R.); 2 Symbiosis Centre for Research & Innovation (SCRI), Symbiosis International Deemed University, Lavale, Pune 412115, Maharashtra, India; 3 Tata Consumer Products Limited, Pune 412111, Maharashtra, India; shashikala.nm@tataconsumer.com; 4 Tata Chemicals Limited, Innovation Centre, Pune 412111, Maharashtra, India

**Keywords:** double-fortified salt, fortification, microencapsulation, iron, anemia, animal model, micronutrients

## Abstract

(1) Background: Iron deficiency anemia is a significant nutritional problem all over the world. Salt formulations supplemented with encapsulated iron and iodine (double-fortified) were tested for their efficacy in managing iron deficiency anemia. In this study, we have checked the effect of these double-fortified salt formulations (iron and iodine) on hemoglobin (Hb) levels in anemic Wistar male rats. (2) Methods: The study was divided into two phases, viz., the development of anemia in the first phase and then the random division of anemic rats into five groups (Groups A to E). These rats were fed with three different salt formulations (Groups A to C); Group D was continued on a low iron diet, and Group E was on a normal pellet diet over a period of 84 days. The level of Hb was tested in each group. (3) Results: The rats in Groups A, B, C, and E recovered from anemia significantly, with higher Hb levels. On day 84, however, the Hb level in Group D continued to decrease. The bodyweight of the rats was not affected in any way. In all of the groups, histopathology examinations in various organs revealed no significant changes. (4) Conclusions: All of the three different salt formulations showed significant recovery in the anemic rats as compared to the rats fed with a normal pelleted diet.

## 1. Introduction

Iron is an essential micronutrient of life. It is required for various physiological and biochemical processes in the human body. Despite the fact that iron deficiency is one of the most common kinds of malnutrition, there is a lack of agreement on the nature and extent of the health repercussions of iron deficiency in populations. The global burden of disease (GBD) was calculated in the year 2000. Iron insufficiency is the ninth most common risk factor in the GBD 2000, accounting for 841,000 deaths and 35,057,000 disability-adjusted life years lost worldwide [1]. According to the WHO report, iron deficiency is 2.5 times greater than iron-deficient anaemia, which is approximately 50% out of two billion anaemic people [2].

Iodine deficiency disorders and iron deficiency anemia (IDA) affect ≥ 30% of the global population. These deficiencies often coexist. Iron and iodine deficiencies result in several nutritional disorders [3,4]; anemia and goiter are two such disorders. Anemia occurs when Hb concentration drops lower than the prescribed level incapacitating the oxygen supply of blood. This is a major public health issue in India and worldwide. Globally, 1.62 billion people get affected, corresponding to 24.8% of the entire population. Preschool-age children have the highest prevalence of anemia (47.4%), whereas the lowest prevalence is in men (12.7%) [5]. In India, anemia is prevalent in all age groups; the most vulnerable groups are infants, young children, adolescents, women of child-bearing age, and those pregnant [6]. Earlier surveys conducted by the National Nutrition Monitoring Bureau (NNMB) and National Family Health Survey (NFHS)-32 showed a high occurrence of anemia [7]. Anemia is often accompanied by goiter or iodine-deficiency disorder; cooperatively, anemia and goiter are serious nutrition-deficiency disorders in India, as in other developing countries [8,9]. There are different factors that contribute to anemia, e.g., deficiency of iron, folic acid, and vitamin B. Currently, inflammation has been considered one of the most important factors, along with iron deficiency [10]. Iron deficiency is the most dominant due to the limited intake of iron-rich foods along with poor bioavailability [11,12]. Iron deficiency anemia [IDA] hinders thyroid metabolism and reduces the effectiveness of iodine prophylaxis [13,14].

Salt has been a preferred channel for iron fortification to combat anemia. A fortunate method of common salt fortification with iron has been developed in India [14,15], and its effectiveness in reducing the universality of anemia has been demonstrated [16]. Double-fortified salt (DFS) is a novel new fortified food product that provides humans with small but crucial amounts of iodine and iron through their diet [17,18].

The DFS developed by the microencapsulation process is a stable formulation that does not develop color. Iodine is stable and has acceptability in different food formulations. Microencapsulation ensures that the added micronutrients remain stable and do not interact or degrade [19].

In infants, deficiency of iron leads to the poor development of the brain [20]. In adults, it leads to poor pregnancy outcomes and decreased immunity levels [21]. To reiterate, about 30% of the world’s population, especially in developing countries, are affected by nutritional disorders due to iron deficiency [22].

The occurrence of iron deficiency anemia can also be controlled by supplementation with medical preparations and dietary diversification. In anemic individuals, iron supplements play a vital role in the rapid improvement of their iron status. As supplementation, electrolytic iron and ferrous sulfate are most commonly used. The bioavailability of non-heme iron is very important, which ranges between 2% and 20%, which is, again, influenced by different inhibiting components in diet [23,24]. The major limitation for reducing the occurrence of iron deficiency anemia with inorganic supplementation is the poor bioavailability.

Humans procure iron from their diet, principally from plant foods and the rest from animal origin foods, either haem or non-haem iron. The majority of dietary iron in Western countries comes from haem iron from meat and meat products. In India, on the other hand, haem iron intake is low, with the majority of people getting their non-haem iron from cereals, pulses, vegetables, and fruits. As a result, Indian cuisine has a low iron level and poor absorption. The present study was undertaken to appraise the bioavailability of iron in rats by using double-fortified salt with iron and iodine.

## 2. Material and Methods

### 2.1. Salt Formulations and Diet

Three salt formulations were prepared by Tata Chemicals Ltd. (TCL), India, using the procured non-iodized Tata Salt available in the market and fortified with 25 ppm Iodine [as potassium iodate]/gram salt and 12 to 14% of iron content/gram salt [as ferrous sulphate] with stearic acid and hydroxypropyl methylcellulose (HPMC). The encapsulation process is developed at TCL. TCL has filed a patent application for this (IN201821011987). Silica was added as an anti-caking agent. The modified AIN-93G Rodent Diet with no added iron was given to the studied animals to induce anemia [Cat No. D03072501N] and it was supplied by Research Diets, Inc., New Brunswick, NJ, USA, [25] Commercially available normal pelleted diets (Nutrimix STD-1020) were procured from Nutrivet Life Sciences, Pune, Maharashtra, India and were fed to the control group (approx. 18 gm/day/rat) as per the guidelines of CPCSEA.

### 2.2. Animal Experiment, Care and Management

Six-week-old male Wistar rats (*n* = 40) were purchased from the National Institute of Bioscience, Pune (a CPCSEA approved breeder). Rats were housed in a quarantine room to acclimate for a week prior to the experiments. The animals were kept in conventional rat cages with corn cob as bedding material [purchased from a local vendor, Pune] and maintained in a controlled environment (22 ± 3 °C and 50% ± 10 relative humidity) with 12 h light/dark cycle.

### 2.3. Clinical Observations of Animals

All animals were observed for general appearance, behavior, and mortality. Rats were also observed for their general signs and condition of the skin, fur, eyes, tail, nose, and abdomen twice daily. Their food and water consumption were observed daily, while their body weights were measured weekly.

### 2.4. Study Design

This is a case-control study. Anemia was developed in male Wistar rats (*n* = 40) by providing them with an iron-deficient diet (Modified AIN-93G Rodent Diet) for 30 days. The composition of the formulations (A, B, C) and the iron-deficient diet is mentioned in Table 1, and the composition of the normal pelleted diet is mentioned in Table 2. Blood samples were collected randomly from the rats and Hb levels were monitored every week. Low Hb level (<10 g/dL) rats were considered anemic [26] and selected after 30 days for the anemic rat groups. Six male rats were kept as a control (Group E), which were provided with a commercially available normal pelleted diet throughout the study. After the development of anemia, these rats (*n* = 8/group) were divided into four different groups (A–D). Groups A, B, and C were provided with a diet containing three different salt formulations. Details of formulated diets are given in Table 1. Rats in Group D were provided with normal pellets after the development of anemia. The Group E rats (*n* = 8) were on the normal pelleted diet during the entire study as a control group. The Hb level was monitored in all groups (A–E) every week for a period of 84 days. The bodyweight of these rats was also recorded weekly. The rats were also observed twice daily for general appearance, behavior, signs of morbidity, and mortality, along with skin, fur, eyes, tail, nose, and abdomen conditions.

Iron-Deficient Diet: composition of iron-deficient diet fed to the rats for the development of anaemia.

### 2.5. Estimation of Hemoglobin [Hb]

Estimation of Hb was done by Drabkin’s method using a kit from SPAN Diagnostics following the instruction given in the manufacturer’s protocol. Briefly, 200 µL of blood was collected from each rat by intra-orbital plexus into a vial containing 15 µL EDTA (10 mg/mL). The test sample was prepared by adding 20 µL of freshly collected rat blood to 5 mL of Drabkin’s reagent. Cyanmethemoglobin was used as a standard, and Drabkin’s solution was used as a blank. Tubes were then incubated at room temperature for 5 min in the dark, and absorbance was recorded at 540 nm against a blank on a UV-Visible spectrophotometer (Thermo Scientific Evolution-201, Shanghai, China).

### 2.6. Histopathology Examination

The rats were euthanized using an overdose of carbon dioxide anesthesia. A histopathological examination was performed as per the standard method by Chaitanya Laboratories [28]. Briefly, the liver, kidney, and heart tissues were collected in a 10% formalin solution. The tissue was processed by dehydrating it in increasing concentrations of alcohol, clarifying it in xylene, and embedding it in paraffin wax. A rotary microtome was used to slice paraffin wax-embedded tissue blocks into 3 μm sections. A hematoxylin and eosin (H & E) stain was used to stain all of the slides. The prepared slides were examined by the pathologist under a microscope (400×, and histopathological alterations were noted. The severity of the observed lesions was recorded as 0 = Not Present, 1 = Minimal (<1%), 2 = Mild (1–25%), 3 = Moderate (26–50%), 4 = Moderately Severe (51–75%), 5 = Severe (76–100%), and the distribution was recorded as focal, multifocal, and diffuse.

### 2.7. Institutional Review Board Statement

The research was carried out in line with the recommendations of the Committee for the Purpose of Control and Supervision of Experiments on Animals [CPCSEA], and the Institutional Animal Ethical Committee [IAEC] of Symbiosis School of Biological Sciences approved this study; IAEC approval number: SSBS/IAEC/06-2016; dated 22 November 2016.

### 2.8. Statistical Analysis

Experimental data, in triplicates of Hb levels during the development of anemia and average bodyweight, were used to calculate the mean and standard deviations in Microsoft Excel 2016. The statistical significance of the data obtained for the Hb levels during the recovery of anemia was determined using a one-way ANOVA GraphPad Prism 5.03 (GraphPad Software, Inc., San Diego, CA, USA). The results were interpreted using a 95% confidence coefficient and 5% significance level.

## 3. Results

### 3.1. Development of Anemia in Rats

**Figure 1:**
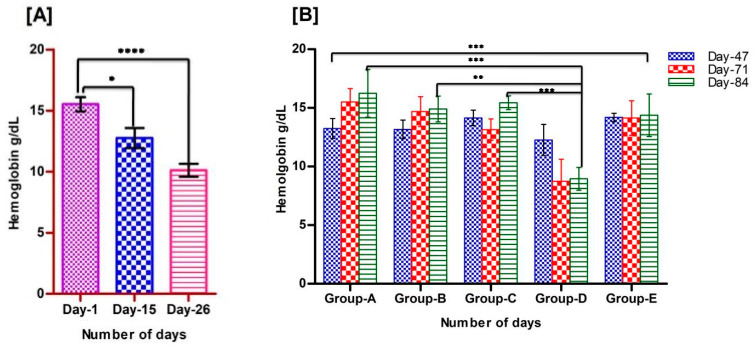
Status of Hb levels in rats, (**A**): Levels of Hb in rats during the development of anemia. Decreased level of Hb was observed on day 26 after feeding an iron-deficient diet, data are presented as mean ± SD, and a two-tailed t-test was performed to see a significant difference; (**B**): Levels of Hb in rats during the regeneration of Hb by feeding diets with fortified salt formulations on day 47, 71, and 84. One-way ANOVA was performed within the groups (Groups A–E) and between the groups (A vs. D, B vs. D, and C vs. D) using GraphPad Prism, and significant results were observed on the recovery of anemia on day 84 (star represents the significance levels; *p* < 0.0001 ****, *p* < 0.001 ***, *p* < 0.01 **, *p* < 0.05 *).

Clinical symptoms of iron deficiency include decreased cognitive abilities, lethargy, and lack of concentration. Lethargy was observed in the rats after the development of anemia. The blood Hb was measured on day 1, day 15, and day 26 (Table 3) to assess the development of anemia. We found that the average level of the blood Hb of the experimental rats decreased significantly to 10.11 g/dL) on day 26 (Figure 1A), which is comparatively lower than the normal range (14–16 g/dL), and, thus, the rats were considered anemic.

### 3.2. Recovery of Animals from Anemia

In the second phase, we formed five groups (A–E), of which three groups (A–C) were fed with three different salt formulations, A-900 ppm (encapsulated ferrous sulphate), B-450 ppm (encapsulated ferrous sulphate), and C-450 ppm (non-encapsulated ferrous sulphate) (Table 1). Group D was continued with a low-iron diet with 4 ppm, whereas Group E was provided with commercially available normal pelleted diet with 450 ppm iron (Table 2). The blood Hb levels of all of the animals in each group were checked on day 47, day 71, and day 84. We found significantly increased Hb levels in rats fed with three different salt formulated diets. The Hb levels were checked randomly for each group weekly. The average Hb level of all of the anemic animals at the beginning of phase II was 10.11 g/dL, which was significantly increased to 16.24, 15.04, and 15.60 g/dL in Groups A, B, and C, respectively, at the end of the phase II study (84 days); whereas the Group D animals remained anemic (Hb 8.98 g/dL) and the Group E animals’, fed with a normal pelleted diet, Hb level was 14.63 g/dL on day 84 (Figure 1B).

### 3.3. Body Weight Analysis

**Figure 2:**
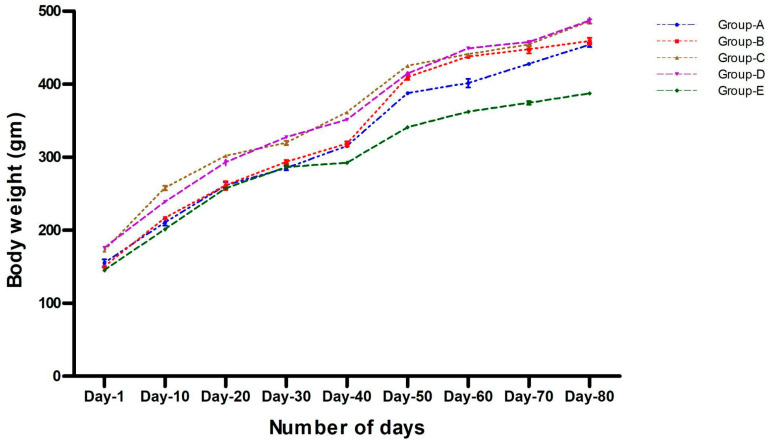
Bodyweight (in grams) of rats in Phase II (recovery period). A significant increase in bodyweight was observed as age increased in all the five experimental groups, whereas there was no significant difference found in the bodyweight of rats on different salt formulations. Data are presented as mean ± SD.

The effect of different iron concentration diets on the bodyweight of the rats was evaluated in this study. We observed that there was no influence of all three iron formulated diets (Groups A–C) on the bodyweight of the rats when compared within the groups (A–D). Similarly, there was no significant difference in the bodyweight of the rats that were given a normal pelleted diet (Group E) when compared with other groups (Groups A–D). The weight gain in all of the groups was comparable to their initial weight. The percentage bodyweight was reported by considering the initial bodyweight as 100% on day 1 (Figure 2).

The percent (%) of bodyweight at day 80: Group “A”, 197.37; “B”, 190.21; “C”, 162.99, “D”, 163.38, and “E”, 178. The % of bodyweight on day one is considered as 100%.

### 3.4. Histopathology

**Figure 3:**
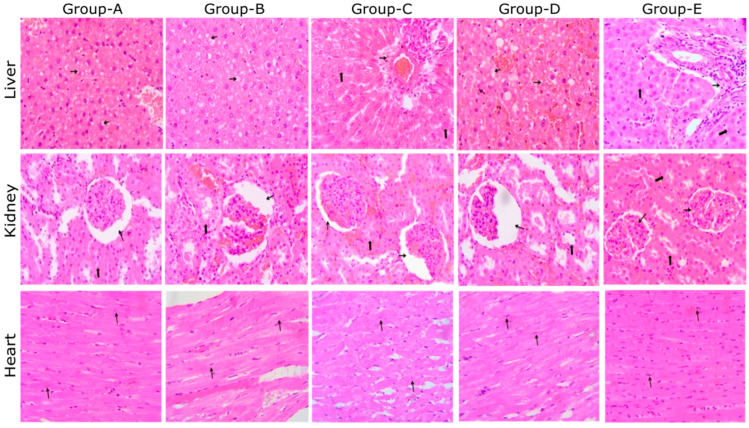
Histopathological analysis of rat liver, kidney, and heart.

Each group’s animals were sacrificed at the end of this study (day 84), and their internal organs (liver, kidney, and heart) were collected to check microscopic changes. The histopathological observations are shown in Figure 3.

Liver: showing cytoplasmic vacuolation of hepatocytes (thin arrow); Groups A, B, and D; showing normal histology, portal triad (thin arrow), and hepatocyte (thick arrow); Groups C and E (H & E, 400×).
Kidney: showing dilated bowman’s space (thin arrow); note the normal tubule (thick arrow); Groups A, B, C, and D; showing normal histology, glomeruli (thin arrow), and tubules (thick arrow); E (H & E, 400×).
Heart: showing normal histology, muscle fiber (thin arrow); A, B, C, D, and E (H & E, 400×).

Histopathological findings revealed a normal architecture of the liver, kidney, and heart tissue among the five experimental groups of rats with different salt formulations in their regular diet after the development of anemia. Sections of the liver in Groups A, B, and D showed cytoplasmic vacuolation of the hepatocytes, whereas, in Groups C and E, there were normal hepatocytes. The kidney sections also showed normal histology of a dilated bowman’s space, glomeruli, and tubules. In the heart sections, a significant difference was not observed; they showed normal histology and muscle fiber.

## 4. Discussion

In this study, we determined the in-vivo effect of different salt formulations on the level of Hb in anemic rats. Our observations indicate that the formulations of iodine and iron played an important role in the recovery of male Wistar rats from iron deficiency anemia. Among other markers, only Hb showed a significant difference when compared with normal male rats, whereas no significant differences were seen in their bodyweight.

Susanti et al. (2017) [29] studied the development of anemia in Wistar rats by providing a low iron diet. They have also found that the administration of low iron in standard food reduces the level of Hb based on the duration in terms of the number of days. They had given a low-iron diet to the animals to induce anemia for 15 days, grouped the animals based on the iron-deficient diet given for a number of days, and they found a reduction in the Hb level in all three groups. The lowest average Hb level (8.5 ± 0.4 g/dL) was observed in the group given a low-iron diet of AIN 93M for 15 days. Xiao et al. (2016) [30] and Zhu et al. (2016) [31] utilized a low-iron diet to induce anemia in the rats for three or eight weeks, resulting in Hb levels of 10 g/dL and 11.9 g/dL, respectively. Yun et al. (2011) [32] used a low-iron AIN 76A diet for four weeks with a Hb level of ≤ 10 g/dL to establish the same model. Following this work, in the present study, hemoglobin levels less than 10 g/dL were considered anemic; these levels were obtained on day 26 after feeding the rats a low-iron diet.

In the previous studies conducted in India by the National Institute of Nutrition (NIN), double-fortified salt (DFS) with iodine and iron showed good potency against iodine deficiency, but there was no overall impact on the Hb concentrations in the 5–15-year-old residential school children [8]. However, we have obtained interesting results in the rat model. The Hb concentrations increased significantly after the administration of iron formulations, where encapsulation material was used to provide a physical barrier around the iron, preventing color changes and potential iodine losses. These findings support Zimmermann et al. (2007) [15], who utilized local salt with 25 g/L potassium iodate and 2 mg Fe (micronized ferric pyrophosphate; mean particle size, 2.5 m/g of salt). There was a notable increase in the bodyweight of all of the animals during their recovery from anemia, fed with diets of all three formulations. Interestingly, significant bodyweight gain was observed during the anemia development stage, as well as during the recovery using all the three salt formulations in the diet. There was no influence of a low-iron diet on the bodyweight of male rats; similar observations were found by Susanti et al. (2017) [29] in female Wistar rats.

The recovered anemic rats (all of the three groups) on fortified diets have not shown any abnormalities in their histopathological examinations (liver, heart, and kidney). In an earlier study by Salaheldin et al. (2016) [33] on a nano iron-fortified diet with an iron concentration of 10 ppm, 30 ppm, and 60 ppm, inflammatory cell infiltration in the liver, kidney, and lungs was observed with a 60 ppm diet. The experimental rats showed no physical or behavioral abnormalities or mortality throughout the study.

In the present study, we found that all three of the formulations from double-fortified salts showed a significant effect on the recovery of anemia. Thus, we conclude that iron deficiencies could be prevented by adding the double-fortified salt into our diet on a regular basis after further clinical studies, which could be helpful for preventing anemia caused due to iron deficiency.

## 5. Conclusions

Double-fortified salt formulations showed a significant effect on anemia in rats. Thus, we conclude that such formulations may be helpful for human applications in improving and maintaining their Hb level.

## Figures and Tables

**Table 1:** Nutritional composition of diets (modified AIN 93G) fed to the rats.

Content	Formulation-A (ppm)	Formulation-B (ppm)	Formulation-C (ppm)	Iron-Deficient Diet (ppm)
Casein	200.00	200.00	200.00	200.00
L-Cystine	3.00	3.00	3.00	3.00
Corn starch	397.486	397.486	397.486	397.486
Maltodextrin 10	132.00	132.00	132.00	132.00
Sucrose	100.00	100.00	100.00	100.00
Avicel PH101	50.00	50.00	50.00	50.00
Soybean oil	70.00	70.00	70.00	70.00
Butylhydroquinone	0.014	0.014	0.014	0.014
Mineral mix S18706	35.00	35.00	35.00	35.00
Ferrous sulphate	900.00	450.00	450.00	0.00
Vitamin mix V10037	10.00	10.00	10.00	10.00
Choline bitartrate	2.500	2.500	2.500	2.500

Formulations details: Formulation A (encapsulated ferrous sulphate) fed to Group A, Formulation B (encapsulated ferrous sulphate) fed to Group B, Formulation C (non- encapsulated ferrous sulphate) fed to Group C.

**Table 2:** Nutritional composition of normal pelleted diet for rats [27].

Sr. No	Ingredients	Percentage
1	Crude protein	18.77
2	Crude fat	4.95
3	Crude fiber	5.30
4	Calcium	0.97
5	Phosphorous	0.68
6	Total ash	5.60
7	Carbohydrates	57.23
8	Metabolizeble energy (kcal/gram)	3.10

**Table 3:** Hemoglobin levels of rats during development of anemia (Phase-I).

Days	Hemoglobin [g/dL]					Mean	SD
Day-1	13.24	15.83	16.25	16.24	16.08	15.52	1.29
Day-15	10.73	13.49	14.95	11	13.64	12.76	1.82
Day-26	10.73	10.96	11.14	9.37	8.37	10.11	1.19

Levels of hemoglobin (g/dL) of rats are presented as mean and SD on day 1, day 15, and day 26.

## Data Availability

All data included in this study will be available by contacting the corresponding author as per the guidelines of the journal under the agreement.
